# Identifying risk factors for late HIV diagnosis and survival analysis of people living with HIV/AIDS in Iran (1987–2016)

**DOI:** 10.1186/s12879-021-06100-z

**Published:** 2021-04-27

**Authors:** Younes Mohammadi, Mohammad Mirzaei, Nasrin Shirmohammadi-Khorram, Maryam Farhadian

**Affiliations:** 1 Department of Epidemiology, School of Public Health, Hamadan University of Medical Sciences, Hamadan, Iran; 2 Modeling of Noncommunicable Diseases Research Center, Hamadan University of Medical Sciences, Hamadan, Iran; 3 Hamadan Health Center, Hamadan University of Medical Sciences, Hamadan, Iran; 4 Department of Biostatistics, School of Public Health, Hamadan University of Medical Sciences, Hamadan, Iran; 5 Research Center for Health Sciences, Department of Biostatistics, School of Public Health, Hamadan University of Medical Sciences, P.O. Box 4171-65175, Hamadan, Iran

**Keywords:** Delayed diagnosis, HIV infections / diagnosis, HIV infections / mortality, Risk factors

## Abstract

**Background:**

Late-diagnosis of HIV is a major challenge for the control and prevention of AIDS in the world. The present study aimed to specify factors associated with the late diagnosis of HIV in Iran from 1987 to 2016.

**Methods:**

In this retrospective cohort study, data for 4402 diagnosed HIV/AIDS patients were extracted from 158 behavioral disease counseling centers of 31 Iranian provinces. We defined late diagnosis as having a CD4 count less than 350 within 3 months after diagnosis. Multiple logistic regression analysis was used to determine the factors influencing late diagnosis. Moreover, we used multivariate Cox regression to assess the association of these factors with the patients’ survival.

**Results:**

In this study, the prevalence of late diagnosis among the patients was 58.2%. People aged 50 years and over (adjusted OR = 3.55), transmission through blood transfusion (adjusted OR = 2.89), co-infection with tuberculosis (adjusted OR = 2.06), and male gender (adjusted OR = 1.38) were the strongest predictors for late diagnosis of HIV. On the other hand, baseline CD4 (adjusted HR = 2.21), people aged 50 and over (adjusted HR = 1.81), male gender (adjusted HR = 1.76), being a widow (adjusted HR = 1.68), people with unknown transmission way (adjusted HR = 18.24), people who inject drugs (adjusted HR = 1.87), diagnosis at previous years (adjusted HR = 2.45) and co-infection with tuberculosis (adjusted OR = 1.77) significantly associated with the survival of patients.

**Conclusion:**

The prevalence of late diagnosis is high among Iranian HIV/AIDS. The risk factors of late diagnoses include being males and aged 50 years and over, transmission through blood transfusion, and co-infection with tuberculosis. Therefore, implementation of screening programs for early diagnosis of HIV these high risk groups is recommended to Iranian health providers and policymakers.

## Introduction

Acquired immunodeficiency syndrome (AIDS) is a potentially life-threatening condition caused by the human immunodeficiency virus (HIV). Globally, about 40 million people are living with this virus, and every year 1.7 million new cases are added to existing cases. Moreover, yearly 700,000 people die from AIDS in the world [1].

Recommended treatment for AIDS is called antiretroviral therapy (ART), which involves taking a combination of HIV medicines [2]. ART aims to reach viral load suppression of HIV in the body, that is, ART reduces the viral load of HIV in the blood to a very low level, and promotes immune system performance, and prevents diseases [3]. Furthermore, ART reduces the probability of HIV transmission to other people when viral load is very high. Despite ART does not cure AIDS, it assists the patients to have a longer and healthier life [4]. Pre-requirement for successful treatment of AIDS is early diagnosis of HIV in the suspected people. Early HIV infection is defined as being diagnosed 6 months after initiation of HIV infection [5]. Despite the importance of early diagnosis of HIV in patients’ treatment, the studies show that late diagnosis has been remained a major problem for the control and prevention of AIDS in the world [6]. Late diagnosis of HIV adversely affects individuals and society by reducing the effectiveness of antiretroviral therapy (ART) and subsequently increasing the risk of morbidity and mortality in the patients. On the other hand, this late diagnosis increases the probability of transmission and spread of the disease in societies, and as a result, control of the disease becomes harder [7, 8].

Studies identified several factors affecting the late diagnosis of HIV. They demonstrated that male gender and older ages significantly increase the probability of late diagnosis [8]. However, the majority of studies concentrated in African or European countries, and there is no much information on the status of diagnosis of HIV in other regions and countries including Iran [9, 10]. Iran, as one of the countries involving in HIV pandemics, is located in Western Asia. Based on the latest statistics in 2018, 61,000 Iranians are living with HIV. Moreover, this report adds that 2600 people died from AIDS-related diseases. Most cases occur in men, ages 15 and 44 years, and drug injection is the most common way for HIV transmission. Accordingly, Iran possesses a concentrated epidemic, which is the largest HIV epidemic among Middle East countries. Moreover, the report shows that 36% of people with HIV aware of their status and only 20% of the patients receive treatment and only 17% of people living with HIV were virally suppressed. Moreover, the statistic states that the majority of the patients (53%) had late HIV diagnosis [11]. However, statistical modeling suggests a number much more than the reported number for Iran, over 75,000 cases and even more [12]. However, due to the religious and cultural limitations in Iran, no comprehensive information is available about AIDS, and especially about the status of late diagnosis of HIV and the related- factors. In this study, we aimed to identify factors that contribute to the late diagnosis of HIV and survival of people with HIV in Iran.

## Methods

The required data for this study was obtained from behavioral disease counseling centers (BDCC), which are established all over Iran. These centers responsible for collecting the demographic and clinical information include age, sex, the date of HIV diagnosis, the mode of HIV transmission, HIV status, the clinical stage of HIV/AIDS, ART reception, CD4 cells count, education level, and marital status of the patients [13]. Inclusion criteria include all HIV-positive patients aged ≥16 years old, which have CD4 cell count within 3 months after diagnosis.

In this study, the late diagnosis was defined as a patient whose first CD4 cell count was less than CD4 < 350 cells/ mm3 within 90 days of diagnosis. Moreover, mortality data were extracted from BDCC. A sub-administration in Behavioral disease counseling centers (BDCC) is responsible for registering of death events for each patient. Therefore, any death event is recorded by BDCC. To assure completeness of death registration, a double check is made with the Iranian Death Registration System (DRS), which is established to register death events in the general population and has appropriate coverage [13], and then the unmatched cases of death were added to death cases of BDCC.

### Statistical analysis

First, we conducted a bivariate analysis to assess the crude association of risk factors with HIV late diagnosis and survival. In this step, any variable whose univariate test has a *p*-value < 0.25 was a candidate for the multivariate analysis. Hosmer and Lemeshow test was applied to check the goodness of fit of the final logistic model, which the model was found fit (*p*–value = 0.57). Also, Odds Ratios (OR) with 95% confidence intervals (95%CI) were used to summarize and describe the strength of association. Moreover, a Cox regression model was used to evaluate the factors affecting the survival of the patients. In the cox model, survival time (in months) was considered from the date of HIV diagnosis to the end of the follow-up period or the occurrence of death. Furthermore, for the patients who had not experienced death or lost to follow-up, the time from the date of HIV diagnosis to the end of the follow-up time was considered as the censored survival time. Moreover, Schoenfeld’s residual test was used to investigate the proportional hazards (PH) assumption of the cox model.

We used the Amelia package in R4.0.3 to impute missing values. The percentage of missing values was 5% overall. Multiple imputations were applied to handle the missing data. In sensitivity analysis, the results of estimating factors associated with late HIV diagnosis and survival analysis were similar in both MI and complete case analyses. Data analyses were performed using Statistical Package for the Social Sciences (SPSS) version 22.0. In all analyses, a *p*-value less than 0.05 was considered statistically significant.

## Results

### Descriptive and bivariate analysis

Data for 4402 patients between 1987 and 2016, whose first CD4 data were measured within 3 months after diagnosis, were retrieved. The mean age of participants was 34.3 ± 10.4 years, and 3030 (68.8%) people were men. Based on the initial CD4 counts, 2564 (58.2%) patients had late-diagnosed and 1838 (41.8%) had the early diagnosis. The mean age of those with a late diagnosis of 37 ± 9.9 years was significantly higher than those timely diagnosed with 31.9 ± 10.4 years (*p* < 0.001) (Table 1).

**Table 1:** Population characteristics of the 4402 of newly diagnosed people living with HIV/AIDS in Iran (1987–2016) stratified by late and early diagnosis

Variable	Late Diagnosis (CD4 < 350^**a**^)		Early Diagnosis (CD4 > 350)		Total
	N	%	N	%	N
**All**	2564	58.2	1838	41.8	4402
**Age (year)**					
< 30	603	41.7	842	58.3	1445
30–40	1167	62.9	689	37.1	1856
40–50	551	70.3	233	29.7	784
> 50	243	76.7	74	23.3	317
**Sex**					
Female	705	51.4	667	48.6	1372
Male	1859	61.4	1171	38.6	3030
**Marital Status**					
Single	774	54.2	655	45.8	1429
Married	1263	59.2	870	40.8	2133
Widow	314	61.8	194	38.2	508
Divorce	168	63.4	97	36.6	265
Unknown	45	67.2	22	32.8	67
**Education**					
Illiterate	201	63.0	118	37.0	319
Primary School	617	60.5	403	39.5	1020
Secondary School	713	56.5	548	43.5	1261
High School	522	56.8	379	43.2	919
Academic	151	55.1	123	44.9	274
Unknown	360	59.1	249	40.9	609
**Job**					
Employed	884	58.1	637	41.9	1521
Unemployed	1009	58.0	731	42.0	1740
Unknown	671	58.8	470	41.2	1141
**Transmission Way**					
Mother to child’s	37	30.3	85	69.7	122
Injecting drug user	1290	61.3	814	38.7	2104
Unprotected sexual	878	53.3	768	46.7	1646
Blood transfusion	11	78.5	4	21.5	14
Unknown	298	57.8	218	42.2	516
**WHO Clinical Stage**					
Stage 1	897	44.9	1100	55.1	1997
Stage 2	371	62.5	223	37.5	594
Stage 3	489	85.0	86	15.0	575
Stage 4	331	89.0	41	11.0	372
Unknown	476	55.1	388	44.9	864
**Year of HIV Diagnosis**					
Before 2006	229	43.2	301	56.8	530
2006–2011	862	62.1	526	37.9	1388
After 2011	1473	59.3	1011	40.7	2484
**TB co-infection**					
No	2374	57.3	1768	42.7	4142
Yes	190	73.1	70	26.9	260

^a^CD4 cell count < 350 cells/mm3 within 91 days of diagnosis

Based on the univariate analysis, late diagnosis was significantly higher in men, older age, people who inject the drug, people who have unprotected sexual contact, divorced persons, and the patients who have tuberculosis co-infection (*p* < 0.05). However, education level and job status were not significantly associated with late diagnosis (*p* > 0.05) (Table 1).

### Multivariate analysis

#### Late HIV diagnosis

The result of the logistic regression model showed that the odds of late-diagnosis for men was 1.4 (95% CI: 1.15–1.67, *p* = 0.001) times that of women. In addition, the odds of late diagnosis in older people significantly were higher than younger people (Table 2).

**Table 2:** Logistic regression analyses of factors associated with late HIV diagnosis in newly diagnosed people living with HIV/AIDS in Iran (1987–2016)

Variable	Unadjusted Odds Ratio	95% CI	***p***-value	Adjusted Odds Ratio	95% CI	***p***-value
**Age (year)**			< 0.001			< 0.001
< 30	Reference			Reference		
30–40	2.36	2.06–2.72		2.06	1.77–2.39	
40–50	3.30	2.74–3.97		2.84	2.33–3.47	
> 50	4.58	3.46–6.07		3.55	2.64–4.80	
**Sex**			< 0.001			0.001
Female	Reference			Reference		
Male	1.50	1.32–1.71		1.38	1.15–1.67	
**Marital Status**			0.001			0.287
Single	Reference			Reference		
Married	1.23	1.07–1.41		1.01	0.85–1.19	
Divorce	1.37	1.11–1.69		1.09	0.87–1.37	
Widow	1.47	1.12–1.92		1.25	0.91–1.72	
Unknown	1.73	1.03–2.91		1.57	0.89–2.78	
**Education**			0.123			0.245
Academic	Reference			Reference		
Illiterate	1.39	0.99–1.93		1.34	0.93–1.91	
Primary School	1.25	0.95–1.63		1.56	0.87–1.55	
Secondary School	1.06	0.82–1.38		1.01	0.74–1.30	
High School	1.07	0.82–1.40		1.05	0.79–1.40	
Unknown	1.18	0.88–1.57		1.14	0.83–1.55	
**Transmission Way**			< 0.001			< 0.001
Mother to child	Reference			Reference		
Injecting drug users	3.64	2.45–5.41		2.20	1.42–3.41	
Unprotected sexual	2.63	1.76–3.91		1.88	1.21–2.91	
Blood transfusion	4.71	3.10–7.29		2.89	1.81–4.61	
Unknown	8.42	2.22–31.96		3.97	0.99–15.83	
**Year of HIV Diagnosis**			< 0.001			< 0.001
After 2011	Reference			Reference		
Before 2006	0.52	0.43–0.63		0.44	0.35–0.54	
2006–2011	1.13	0.98–1.29		1.15	0.99–1.33	
**TB co-infection**			< 0.001			< 0.001
No	Reference			Reference		
Yes	2.02	1.53–2.68		2.06	1.52–2.78	
**Job**			0.902			–
Employed	Reference			–		
Unemployed	0.99	0.86–1.14		–	–	
Unknown	1.03	0.88–1.20		–	–	

In the transmission mode, the strongest association with late diagnosis belongs to blood transfusion (OR = 2.89, 95%CI; 1.81–4.61), and the weakest association belongs to unprotected sex (OR = 1.88, 95% CI; 1.21–2.91) (Table 2).

TB co-infection was significantly associated with late diagnosis. People with TB significantly had higher odds for late HIV diagnosis than people without TB (OR = 2.06, 95%CI; 1.52–2.78).

Marital status and education level had not a significant association with late diagnosis (*p* > 0.05) (Table 2).

#### Survival analysis

**Fig. 1:**
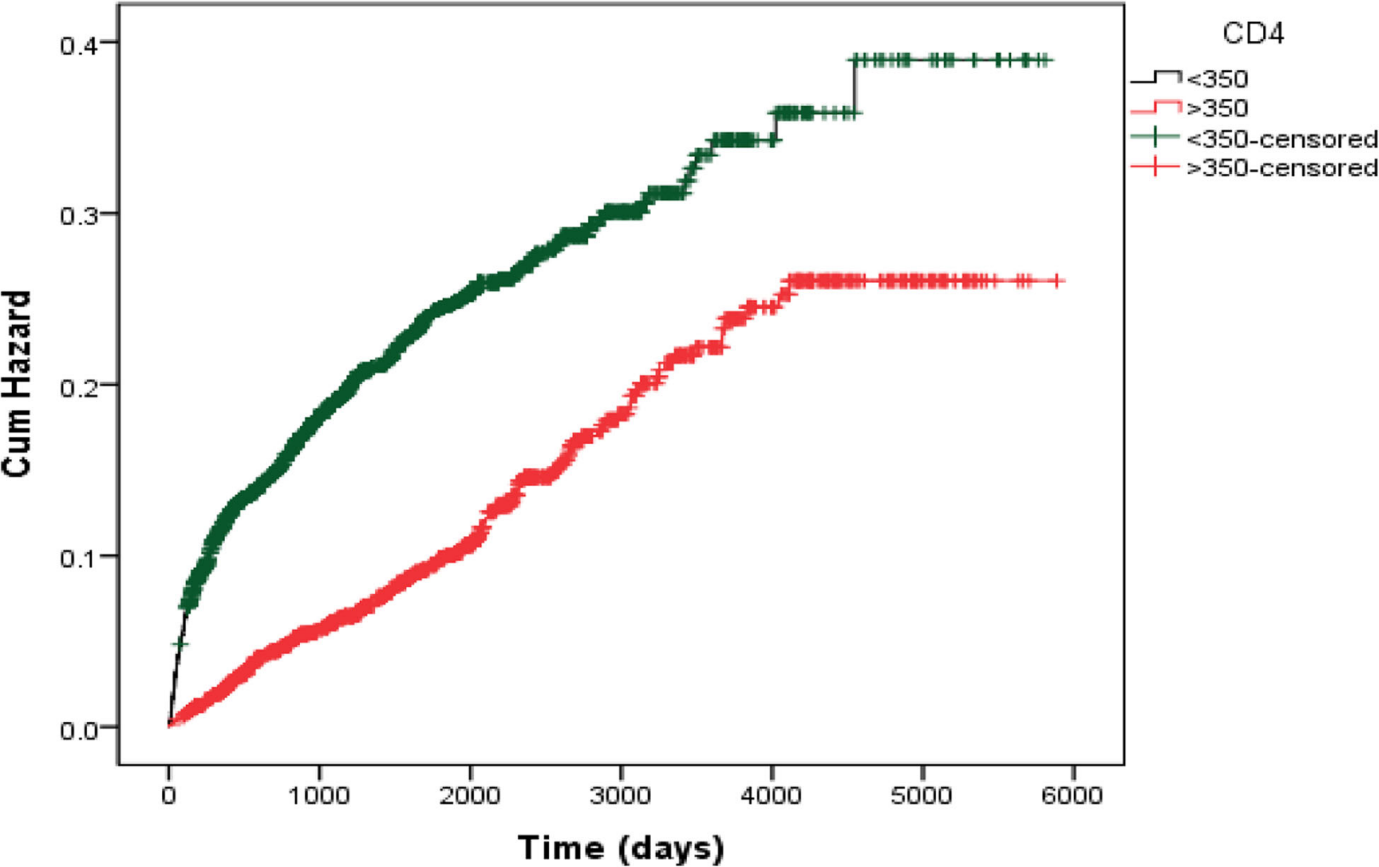
Kaplan-Meier curves of newly diagnosed people living with HIV/AIDS in Iran (1987–2016) defined by CD4 status within 91 days of diagnosis

There was a statistically significant difference in the median survival time (day) between patients with early diagnosis (5009.53 ± 63.84) and late diagnosis (4404.90 ± 68.37). The Kaplan Meier plot for these two groups is shown in Fig. 1. Out of 674 patients who died, 492 (73%) had the late diagnosis, while 182 (27%) had the early diagnosis. Despite in total, 66.9% received ART, in subgroups, 31% of patients who died and 33.4% patients who survive received ART (Table 3).

**Table 3:** Cox regression analyses of factors associated with survival in newly diagnosed people living with HIV/AIDS in Iran (1987–2016)

Variable	Unadjusted Hazard Rate	95% CI	***p***-value	Adjusted Hazard Rate	95% CI	***p***-value
**Baseline CD4**			< 0.001			< 0.001
> 350	Reference			Reference		
< 350	2.25	1.89–2.67		2.21	1.84–2.64	
**Age (year)**			< 0.001			< 0.001
< 30	Reference			Reference		
30–40	1.34	1.10–1.62		1.01	0.83–1.23	
40–50	1.95	1.57–2.43		1.36	1.08–1.72	
> 50	2.29	1.73–3.04		1.81	1.33–2.88	
**Sex**			< 0.001			< 0.001
Female	Reference			Reference		
Male	3.20	2.53–4.04		1.76	1.28–2.42	
**Marital Status**			< 0.001			0.002
Single	Reference			Reference		
Married	0.65	0.55–0.78		0.86	0.71–1.04	
Divorce	1.23	0.99–1.53		1.08	0.86–1.35	
Widow	0.87	0.63–1.22		1.68	1.14–2.47	
Unknown	1.30	0.74–2.26		1.45	0.82–2.55	
**Education**			< 0.001			0.036
Academic	Reference			Reference		
Illiterate	2.27	1.38–3.45		1.47	0.88–2.45	
Primary School	2.09	1.34–3.29		1.49	0.93–2.38	
Secondary School	1.93	1.23–3.02		1.30	0.82–2.07	
High School	1.41	0.88–2.24		1.09	0.67–1.76	
Unknown	1.58	0.98–2.54		1.08	0.66–1.76	
**Transmission Way**			< 0.001			< 0.001
Mother to child	Reference			Reference		
Injecting drug users	3.75	1.77–7.90		1.87	0.86–4.08	
Unprotected sexual	1.13	0.53–2.43		1.01	0.45–2.24	
Blood transfusion	1.65	0.75–3.66		1.08	0.46–2.40	
Unknown	17.25	11.01–32.45		18.24	7.06–37.09	
**Year of HIV Diagnosis**			< 0.001			< 0.001
After 2011	Reference			Reference		
2006–2011	3.08	2.51–3.78		2.45	1.97–3.04	
Before 2006	1.42	1.17–1.72		1.22	0.99–1.49	
**TB co-infection**			< 0.001			< 0.001
No	Reference			Reference		
Yes	2.82	2.28–3.49		1.77	1.42–2.21	
**Job**			0.269			
Employed	Reference			Reference		
Unemployed	1.13	0.93–1.38		–	–	–
Unknown	0.99	0.82–1.21		–	–	–

In multivariate analysis, late diagnosis has a significant impact on the survival of patients. The odds of dying in people with late diagnosis were approximately two times higher than those with early diagnosis (Table 3). Furthermore, people aged 50 years and over are significantly less likely to survive than those aged under 30 years with HR = 1.81 (95% CI: 1.33–2.88). The odds of dying in men were 1.76 (95% CI: 1.28–2.42) times higher than women. The widows had higher odds for dying than the singles (1.68; 95% CI: 1.14–2.47). In addition, people with TB are at a higher risk of death compared to those without TB infection, with an HR = 1.77 (95% CI: 1.42–2.21). People diagnosed between 2006 and 2011 were significantly more at risk of death than those diagnosed after 2011 with a 2.45 (95% CI: 1.97–3.04) times higher odds of dying compared to after 2011. However, job status has no significant effect on the survival of patients (*p* > 0.05) (Table 3).

## Discussion

This is the first study investigating the status of late HIV diagnosis and its factors in Iran. We found that late diagnosis was prevalent among Iranian patients (53%). Existing evidence show that prevalence of late HIV diagnosis is high in other studies. Accordingly, the reported prevalence ranged from 25% in Columbia to 70.1% in China [14, 15]. It is a major concern for the country because people with late HIV diagnosis receive ART late and, as a result, loses opportunities for counseling, education, and substance abuse treatment, resulting in the spread of HIV in the community. Evidence confirms that ART has the maximum effect on viral load and mortality rate from AIDS in the early stages of the disease. Therefore, ART is recommended to be initiated for all HIV-positive people immediately after diagnosis [16].

Moreover, we found that people who inject drugs (PWID) had the highest risk of late diagnosis. Like our study, a study conducted in China show that odds of late diagnosis in PWID was two times higher than other people [17]. Due to the severe social discrimination and stigma against both drug abuse and HIV in Iran, PWID avoid seeking medical services, including tests for HIV diagnosis. The literature has well established that ART is less prevalent in PWID than people who do not inject drugs. Additionally, in counties with free health services for HIV patients, medical care utilization is very low due to the patients’ social situations and family problems [18, 19].

In this study, the probability of late diagnosis was significantly greater in Iranian men than women. Other studies confirmed that male gender is a major risk factor of risky behaviors such as HIV late diagnosis [20]. For example, Rice et al., Sun et al. and Agaba et al. suggested that male gender is significantly associated with a higher risk of late HIV diagnosis [21–23]. This result could be attributed to two reasons. First, most PWID are men on the one hand, and late diagnosis is highly prevalent among PWID on the other hand. Therefore, men PWID are dominant in the AIDS population and have a higher frequency of late diagnosis than women [24]. Second, studies show that men generally are less likely to seek out health care than women. These studies have highlighted the fear of developing a disease as a significant barrier to seek medical care in men [25].

We found that the risk of late diagnosis was higher in older patients than in younger ones. A review of the literature also confirms that older age is the predominant predictor of late presentation of HIV [16, 22, 23, 26]. Studies show that older patients have a low-risk perception relative to HIV, and therefore are less likely to be tested for HIV [8]. Late diagnosis is a significant factor of mortality in older people, and in fact, these groups obtain the most significant benefit from ART compared to other age groups [27]. Low education and knowledge and low risk perception about the disease have been mentioned as the main reasons for late diagnosis in older people. Low education is also associated with low socioeconomic status, affecting health and medical services [28, 29].

Another identified risk factor of late HIV diagnosis was co-infection with TB. Gesesew et al. showed that HIV people with TB co-infection were about 2 times at risk of late HIV diagnosis [9].

This study showed that the prevalence of late HIV diagnosis was the lowest in mother-to-child transmission compared to the other transmission modes. Currently, a program called PMTCT (prevention of mother-to-child transmission) is being implemented [30]. All mothers are tested for HIV during pregnancy and are provided with HIV counseling. Preventive treatment should be given to the baby if the mother’s test is positive. All infants born to HIV-positive mothers are also screened for HIV prophylaxis after birth and HIV. If a baby is infected with HIV, they will receive the same care and treatment services as other HIV-positive patients for the rest of their lives. This program aims to reduce the rate of mother-to-child transmission, one of the goals of the HIV prevention and control program recommended to countries by WHO and UNAIDS [31]. This study confirms the effectiveness of PMTCT on early HIV diagnosis. Therefore, it is recommended to improve the program and expand it to all regions of Iran.

This study’s results provide implications for Iranian policymakers and health providers. First, the percentage of late diagnosis is high and require more attention. Second, policymakers should design programs to perform screening among high-risk subgroups including PWID and FSWs. These groups should be encouraged to seek earlier diagnosis and treatment. Iranian policymakers should conduct scaling up HIV testing, making a considerable percentage of individuals infected with HIV receive HIV test [32]. Moreover, we recommend performing the mass screening of HIV and expanding counseling centers for high-risk groups, including FSWs and PWID. Furthermore, the increased coverage of ART is recommended to improve patient survival.

We used registered data for this study as one of the study’s limitations. Moreover, we did not measure some critical variables in late diagnosis and survival. Therefore, we could not assess their effects on late diagnosis or survival and could not control their confounding effects. Furthermore, the precision and validity of our retrospective study’s data are questionable because the data were not collected for research objectives. Accordingly, the retrospective studies may potentially produce selection bias or information bias, and therefore, may distract the final results. Additionally, registered data lack data verification and do not have complete data/follow-up.

## Conclusion

The prevalence of late HIV/AIDS diagnosis is very high among Iranians. For breaking the disease transmission chain, early HIV diagnosis through facilitating access to HIV testing for PWID by incorporating community-based outreach, drug abuse treatment, and syringe exchange programs is recommended. Moreover, to improve survival of the patients, and decelerate the progression of the disease, supplying ART for all patients with AIDS is crucial.

## Data Availability

The dataset analyzed during the current study is not publicly available due to the sensitivity of the subgroup (HIV/AIDS patients) studied, but is available from the corresponding author on reasonable request.

## Abbreviations

CDC: Centers for Disease Control and Prevention
ART: Antiretroviral Therapy
HAART: Highly Active Antiviral Therapy
BDCC: Behavioral disease counseling centers
DRS: Death Registration System
PWID: People who inject drugs
